# Nasotracheal intubation and male sex as independent risk factors for ICU-acquired sinusitis in neurological patients: A retrospective cohort study

**DOI:** 10.1097/MD.0000000000046481

**Published:** 2025-12-26

**Authors:** Yubin Xue, Lin Guan, Dong Ma, Yaqiang Zhang, Chao Dong, Jianan Ma, Jianwei Wu

**Affiliations:** a Department of Otorhinolaryngology, Shizuishan First People’s Hospital, Ningxia, China; b Department of Neurology, Beijing Tiantan Hospital, Capital Medical University, Beijing, China.

**Keywords:** nasogastric tube, nasotracheal intubation, neurological intensive care unit, pneumonia, sinusitis

## Abstract

Hospital-acquired sinusitis is an underrecognized complication in critically ill neurological patients and may contribute to secondary infections and poor outcomes. This study aimed to evaluate the incidence and progression of sinusitis in the neurological intensive care unit (NICU) and to identify independent clinical risk factors. We conducted a single-center retrospective study of 250 patients admitted to the NICU at Beijing Tiantan Hospital between December 2020 and December 2021. Serial cranial computed tomography (CT) scans were evaluated using a modified Lund–MacKay scoring system. Sinusitis progression was defined as any increase in CT score from baseline. Univariable and multivariate logistic regression analyses were performed to determine independent predictors of sinusitis progression. Among the 250 patients (median age 63 years; 66% male), 58% had sinus opacification on admission. Progressive sinusitis was significantly associated with male sex (OR = 1.916, 95% confidence intervals: 1.002–3.666, *P* = .049) and nasotracheal intubation (OR = 5.792, 95% confidence intervals: 1.653–20.297, *P* = .006) as independent risk factors. Other factors, including hospital stay, diabetes, and pneumonia, were not significant in multivariate analysis. Nasotracheal intubation and male sex were independently associated with radiologic progression of ICU-acquired sinusitis in neurological patients. Implications for future research: prospective multicenter studies are warranted to test prevention and care protocols.

## 1. Introduction

Hospital-acquired sinusitis is a clinically significant but often underdiagnosed complication in intensive care unit (ICU) patients, contributing to fever of unknown origin, secondary infections, and increased healthcare costs.^[1–3]^ It has been closely linked to hospital-acquired pneumonia and can adversely affect patient outcomes. Accurate diagnosis is challenging because sedation, impaired consciousness, and limited ability to report symptoms frequently prevent ICU patients from undergoing adequate physical examinations, leading to delayed recognition or underestimation of this condition.^[4,5]^

The reported incidence of ICU-acquired sinusitis varies widely, ranging from 1.5% to nearly 100%, depending on the study population, diagnostic methods, and criteria applied.^[6]^ This variability highlights the need for standardized and objective diagnostic approaches, particularly in high-risk ICU subgroups. Neurological intensive care unit (NICU) patients are especially vulnerable, as they often require prolonged sedation, mechanical ventilation, and invasive procedures, all of which may predispose them to sinus inflammation.

Routine cranial computed tomography (CT) imaging in NICU patients provides a unique opportunity to detect sinus pathology early. CT offers superior visualization of sinus anatomy and disease extent^[7]^ and allows objective scoring using validated systems such as the modified Lund–MacKay scale. This enables longitudinal monitoring of sinusitis progression and facilitates the identification of potential risk factors in critically ill patients.

This study aimed to systematically evaluate the incidence and radiologic progression of sinusitis in NICU patients and to identify clinical factors associated with its development. By determining independent risk factors for sinusitis progression, we seek to provide evidence that can guide early recognition and inform future preventive strategies of sinus-related complications in the critical care setting.

## 2. Methods

### 2.1. Study design and participants

This retrospective cohort study included patients admitted to the NICU of Beijing Tiantan Hospital, Capital Medical University, between December 2020 and December 2021. A complete 1-year observation period was selected to minimize potential seasonal effects on the incidence of sinusitis. This study was conducted and reported in accordance with the strengthening the reporting of observational studies in epidemiology (STROBE) guidelines for observational studies, and the completed STROBE checklist is provided as a supplementary file. The primary objective was to determine the incidence and radiologic progression of sinusitis; secondary objectives included baseline prevalence, temporal changes in Lund–MacKay scores, and associated clinical risk factors.

Inclusion criteria were:patients who underwent cranial CT prior to admission; and patients who received at least 1 head CT scan within 14 days after NICU admission.

Exclusion criteria were: history of facial or skull base fractures; prior nasal or skull base surgery; malignant tumors of the nasal cavity or paranasal sinuses; ICU stay of <2 nights; and hospitalization prior to NICU admission.

### 2.2. Imaging evaluation

Sinusitis was evaluated using the modified Lund–MacKay CT scoring system proposed by the University of Miami.^[8]^ All CT images were reviewed by the same physician, who scored each sinus – anterior ethmoid, posterior ethmoid, frontal, sphenoid, and maxillary – on both sides. Scoring criteria were: 0 = normal (no soft tissue density); 1 = 1–33% opacification; 2 = 34–66% opacification; 3 = 67–99% opacification; 4 = 100% opacification. The total score per side was calculated out of a maximum of 20 points. The presence and laterality (left/right) of nasotracheal intubation (NTT) and nasogastric tube (NGT) at the time of CT imaging were also recorded. Baseline CT was obtained on NICU admission; subsequent scans were performed as clinically indicated and grouped into 3 predefined intervals (days 3–7, 8–14, >14). The highest score in each interval was recorded.

### 2.3. Definition of sinusitis progression

The first Lund–MacKay score obtained on the day of NICU admission was defined as the baseline. The highest scores during 3 time intervals – days 3 to 7, days 8 to 14, and beyond day 14 – were compared to baseline. Sinusitis progression was defined as any increase in the Lund–MacKay score during hospitalization.

### 2.4. Data collection and variables

Demographic and clinical variables collected included age, sex, length of hospital stay, admission diagnosis (e.g., cerebral infarction, cerebral hemorrhage), type of surgical intervention (e.g., craniotomy or endovascular procedure), history of diabetes mellitus, pneumonia during hospitalization, antibiotic use, and the presence and laterality of NTT and NGT.

### 2.5. Statistical analysis

All statistical analyses were performed using SPSS software (version 25.0, IBM Corporation, Armonk). Sinusitis progression was used as the dependent variable. Normality of continuous variables was assessed using the Shapiro–Wilk test. Normally distributed variables are presented as mean ± SD, and skewed variables as median (IQR 25th–75th percentile). Patients were grouped according to sinusitis progression (yes vs no). Group comparisons were made using Student *t* test or Mann–Whitney *U* test for continuous variables and χ^2^ or Fisher exact test for categorical variables. Variables with *P* < .10 in Univariable analysis, along with clinically relevant factors, were entered into a multivariable logistic regression model. Multicollinearity was assessed by variance inflation factor (VIF < 5). Model parsimony was ensured by maintaining adequate events-per-variable ratios to avoid overfitting. Adjusted odds ratios with 95% confidence intervals were reported. A 2-tailed *P* < .05 was considered statistically significant. Variables entered into the multivariable model were prespecified as: sex, age, length of hospital stay, pneumonia, diabetes, antibiotic use, NGT, NTT, craniotomy, and endovascular procedures. Analyses were performed on complete cases; no imputation was performed.

### 2.6. Ethics statement

This retrospective study was approved by the Ethics Committee of Beijing Tiantan Hospital, Capital Medical University (approval no. [KY2024-020-03]). Informed consent was waived due to the observational nature of the study and anonymization of patient data. All methods followed the Declaration of Helsinki and institutional ethical guidelines.

## 3. Results

### 3.1. Patient characteristics

**Figure 1.**
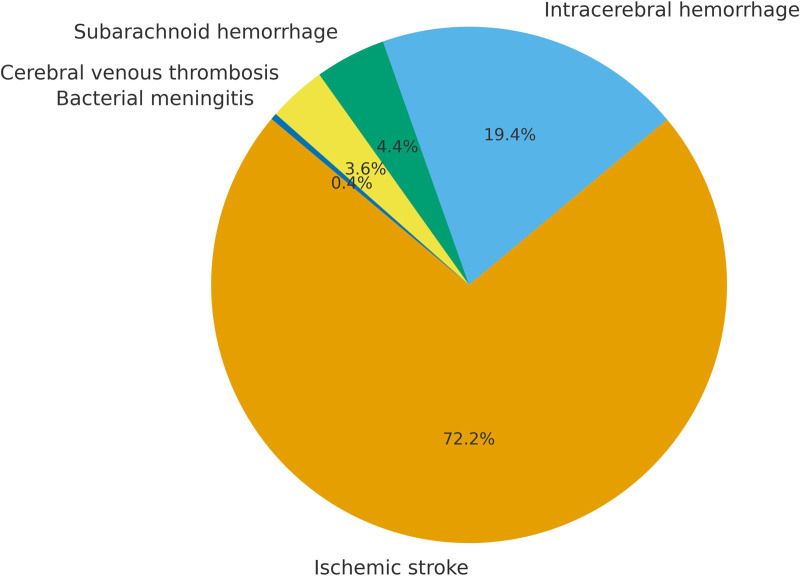
Distribution of primary diagnoses among neurological ICU patients (N = 250). ICU = intensive care unit.

A total of 250 patients admitted to the NICU were included in the final analysis. The median age was 63.0 years (IQR 50.0–72.0), and 165 patients (66.0%) were male. The median length of hospital stay was 12.0 days (IQR 8.0–17.0). The most common admission diagnosis was cerebral infarction (71.6%), followed by cerebral hemorrhage (19.2%) and subarachnoid hemorrhage (4.4%). The distribution of primary diagnoses is shown in Figure 1. Surgical interventions included open cranial surgery (19.2%) and endovascular procedures (50.4%). Details of surgical interventions are provided in Table S1 (Supplemental Digital Content, https://links.lww.com/MD/Q891).

In terms of supportive interventions, NGTs were placed in 82.8% of patients, and NTT was performed in 19.2%. Additionally, 76.8% of patients were diagnosed with pneumonia during hospitalization, and 28.0% had a history of diabetes mellitus. Antibiotic therapy was administered in 70.0% of cases. Baseline head CT was performed in all patients; follow-up CTs were obtained in 209 patients (days 3–7), 155 patients (days 8–14), and 42 patients (>14 days). Detailed baseline characteristics are presented in Table 1.

**Table 1:** Baseline characteristics of the study cohort (N = 250).

Variable	Value
Age, yr	63.0 (IQR 50.0–72.0)
Hospital stay, d	12.0 (IQR 8.0–17.0)
Male sex, n (%)	165 (66.0)
Pneumonia, n (%)	192 (76.8)
Diabetes, n (%)	70 (28.0)
Antibiotics, n (%)	175 (70.0)
NGT present, n (%)	207 (82.8)
NTT present, n (%)	48 (19.2)
CT scans per patient, median (IQR)	2 (2–3)

CT = computed tomography, IQR = interquartile range, NGT = nasogastric tube, NTT = nasotracheal intubation.

### 3.2. Lund–MacKay score progression over time

Baseline head CT was available in 250 patients; follow-up CTs were available in 209 (days 3–7), 155 (days 8–14), and 42 (>14 days). Median modified Lund–MacKay scores increased from 0.0 (IQR 0.0–2.0) at baseline to 5.0 (2.0–9.0) on the left and 3.5 (1.0–7.0) on the right after > 14 days. On admission, 58.0% of patients had evidence of paranasal sinus opacification on CT scans, with a median Lund–MacKay score of 0.0 on both sides. Over time, CT imaging revealed a progressive increase in sinus inflammation scores, particularly beyond day 7. The left-sided median score increased from 0.0 to 5.0, and the right-sided score rose from 0.0 to 3.5 after 14 days of hospitalization. These temporal changes are summarized in Table 2.

**Table 2:** Lund–MacKay score progression over time.

Time period	Left score (median [IQR])	Right score (median [IQR])
Day 1	0.0 (0.0–2.0)	0.0 (0.0–2.0)
Days 3–7	3.0 (0.0–5.0)	2.0 (0.0–6.0)
Days 8–14	3.0 (1.0–8.0)	4.0 (1.0–8.0)
>Day 14	5.0 (2.0–9.0)	3.5 (1.0–7.0)

Values are presented as median (IQR 25th–75th percentile).

IQR = interquartile range.

**Figure 2.**
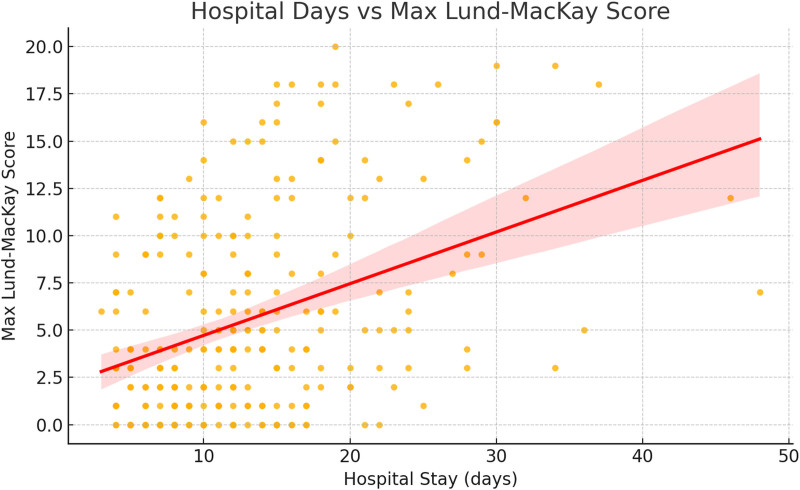
Association between hospital length of stay and maximum Lund–MacKay CT score. Scatter plot with linear regression line illustrating an apparent positive association between hospital stay duration and maximum sinusitis severity score during admission. CT = computed tomography.

A scatter plot analysis further demonstrated a positive correlation between hospital length of stay and the maximum Lund–MacKay score, suggesting that prolonged hospitalization may contribute to sinusitis progression (Fig. 2).

### 3.3. Lund–MacKay score and surgical intervention

**Figure 3.**
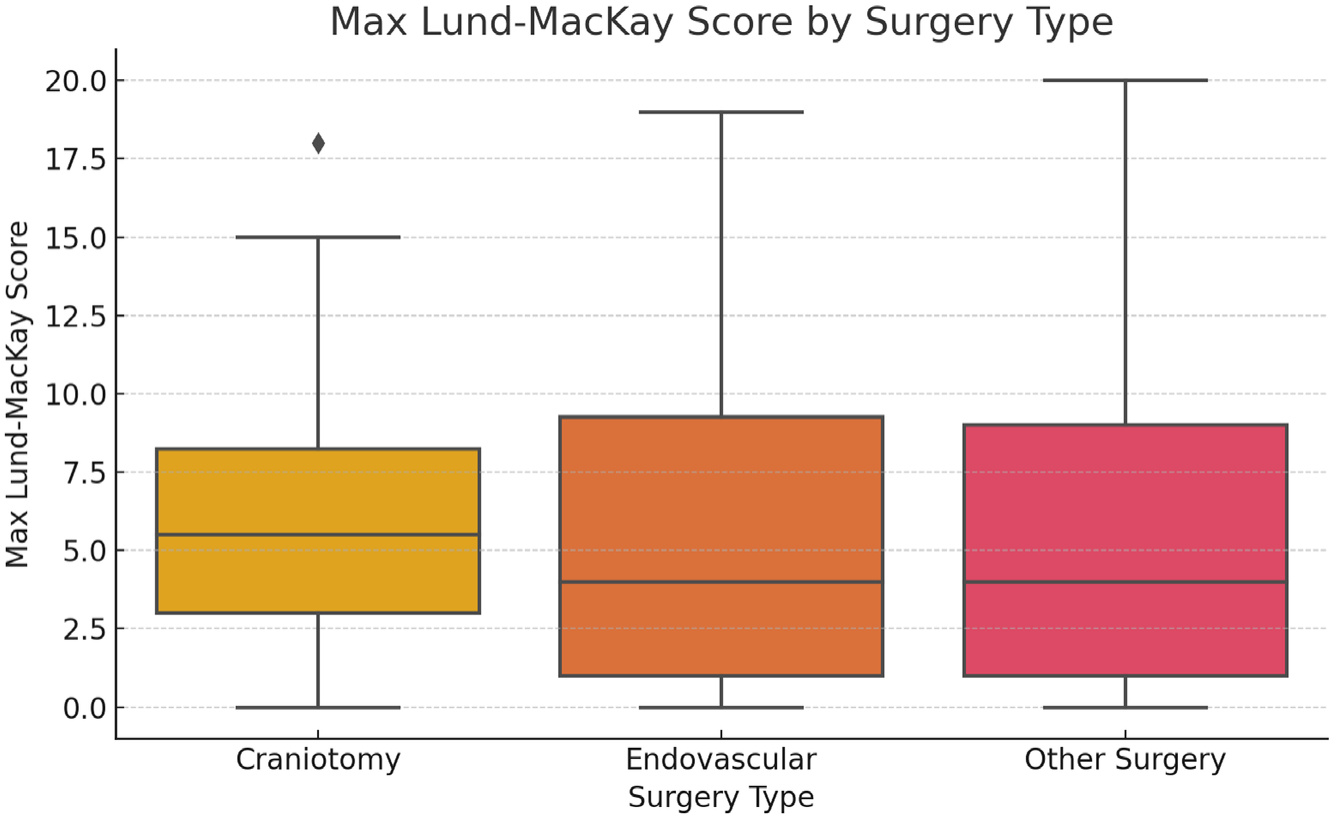
Maximum Lund–MacKay CT scores stratified by surgical intervention type. Boxplot comparing the severity of sinusitis among patients undergoing different types of surgery: craniotomy, endovascular procedures, or other treatments. Differences suggest surgical invasiveness may influence sinus inflammation. CT = computed tomography.

Patients were stratified by type of surgical intervention. As shown in Figure 3, those who underwent craniotomy had higher maximum Lund–MacKay scores compared to those receiving endovascular or other surgical treatments, indicating that surgical invasiveness may influence the severity of sinus inflammation.

### 3.4. Risk factors for sinusitis progression

In univariable logistic regression, male sex (OR = 2.154, *P* = .008), longer hospital stay (OR = 1.105 per day, *P* < .001), craniotomy (OR = 2.170, *P* = .043), pneumonia (OR = 4.101, *P* < .001), diabetes (OR = 1.950, *P* = .048), antibiotic use (OR = 3.760, *P* < .001), NGT placement (OR = 4.327, *P* < .001), and NTT use (OR = 7.954, *P* = .001) were significantly associated with sinusitis progression. In addition, when patients were grouped by progression status (yes vs no), those with progression had longer hospital stays (13.0 [9.0–18.0] vs 9.5 [7.0–14.0] days; *P* < .001) and higher proportions of nasotracheal intubation (25.6% vs 4.1%; *P* < .001), nasogastric tubes (89.2% vs 67.6%; *P* < .001), pneumonia (84.7% vs 58.1%; *P* < .001), diabetes (31.8% vs 18.9%; *P* = .038), and antibiotic use (78.4% vs 50.0%; *P* < .001). Male sex was also more frequent among those with progression (71.0% vs 54.1%; *P* = .010), whereas age did not differ materially (63.0 (52.0–72.0) vs 61.5 (47.0–72.8) years; *P* = .510). Overall, progression occurred in 176/250 patients (70.4%). Detailed results are presented in Table 3.

**Table 3:** Univariable logistic regression analysis.

Variable	OR	95% CI	*P*-value
Sex (male vs female)	0.464	0.264–0.815	.008
Age (continuous)	1.012	0.993–1.030	.212
Hospital stay (continuous)	1.105	1.050–1.162	<.001
Primary diagnosis: Cerebral infarction	0.839	0.554–1.268	.404
Pneumonia	0.244	0.131–0.453	<.001
Diabetes	0.513	0.264–0.995	.048
Antibiotics	0.266	0.149–0.476	<.001
Open cranial surgery vs Other	0.683	0.470–0.992	.045

Values are odds ratios (OR) with 95% confidence intervals (CI) and Wald *P* values. Reference category for surgical treatment: other. Endovascular treatment was analyzed but did not yield statistically significant associations and is therefore not tabulated.

**Figure 4.**
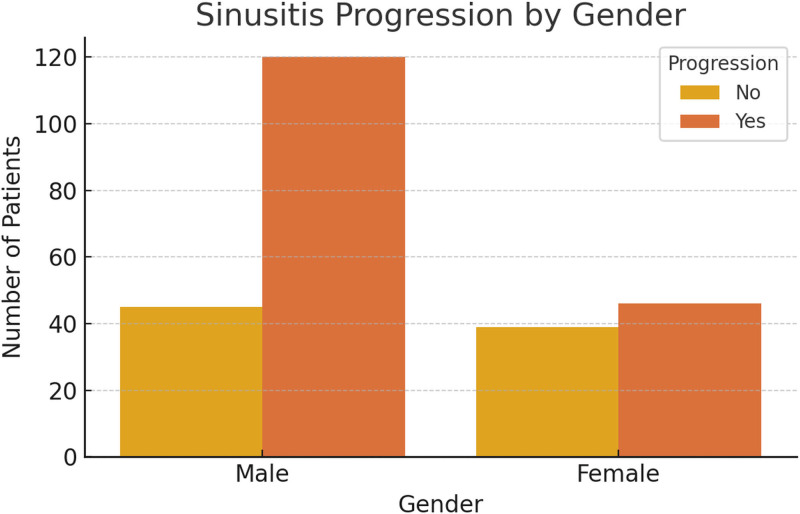
Distribution of sinusitis progression by gender. Bar chart showing the number of male and female NICU patients with or without radiological sinusitis progression. Males demonstrated a higher proportion of sinusitis aggravation compared to females. NICU = neurological intensive care unit.

**Figure 5.**
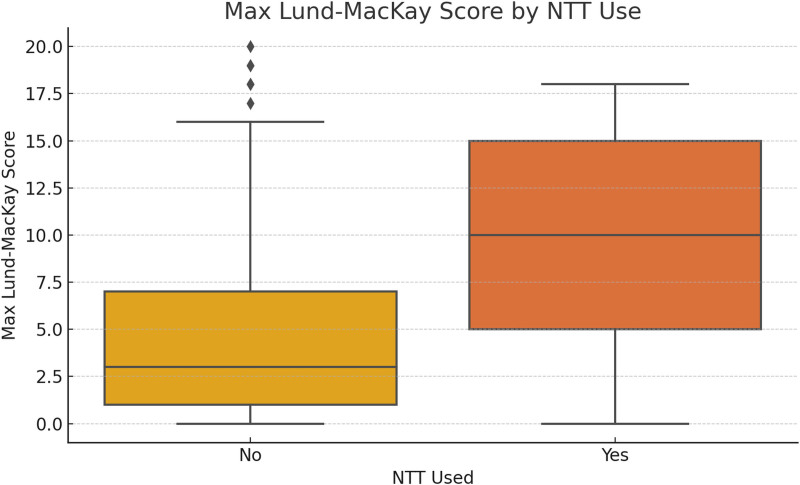
Maximum Lund–MacKay CT scores in patients with and without NTT. Boxplot illustrating that NICU patients who underwent NTT had significantly higher maximum Lund–MacKay scores, indicating greater sinus inflammation. CT = computed tomography.

In the sex subgroup analysis, male patients exhibited a significantly higher proportion of sinusitis progression compared to female patients (Fig. 4). Furthermore, patients who underwent NTT demonstrated substantially higher maximum Lund–MacKay scores, as illustrated in Figure 5.

### 3.5. Independent predictors of sinusitis progression

Multivariate logistic regression analysis was performed to identify independent risk factors. As shown in Table 4, male sex (OR = 1.916, *P* = .049) and NTT use (OR = 5.792, *P* = .006) remained significantly associated with sinusitis progression after adjusting for confounders. Other variables, including hospital stay duration, pneumonia, and diabetes, were not statistically significant in the multivariate model.

**Table 4:** Multivariate logistic regression analysis.

Variable	OR (95% CI)	*P*-value
Gender (male vs female)	1.916 (1.002–3.666)	.049
Age (yr)	1.005 (0.983–1.027)	.654
Hospital stay (d)	1.050 (0.996–1.106)	.070
Surgery: craniotomy	1.796 (0.634–4.940)	.239
Surgery: endovascular	1.014 (0.494–2.078)	.440
Pneumonia (yes vs no)	1.850 (0.709–4.826)	.209
Diabetes (yes vs no)	1.685 (0.796–3.569)	.173
Antibiotic use (yes vs no)	1.283 (0.506–3.252)	.599
NGT (yes vs no)	1.894 (0.810–4.429)	.141
NTT (yes vs no)	5.792 (1.653–20.297)	.006

CI = confidence interval, OR = odds ratio, NGT = nasogastric tube, NTT = nasotracheal intubation.

## 4. Discussion

In this retrospective cohort study of 250 NICU patients, we observed a high incidence of radiologically confirmed sinusitis, with clear progression over the course of hospitalization. Multivariate analysis identified nasotracheal intubation (NTT) and male sex as independent risk factors for sinusitis progression. These findings highlight the importance of early recognition, and implications for preventive strategies should be addressed in future research.

Diagnosing sinusitis in ICU patients is inherently challenging because of impaired consciousness, communication barriers, and multiple comorbidities. Routine physical examination is often limited, making imaging essential for detection. Our results support previous studies demonstrating that CT-based evaluation allows objective, longitudinal assessment of sinus inflammation and can reveal disease progression that might otherwise go unnoticed.^[1,2,4,6,9–11]^ The observed temporal increase in Lund–MacKay scores, especially after 7 to 14 days of ICU stay, aligns with the pathophysiology of nosocomial sinusitis.

NTT emerged as the strongest independent risk factor for sinusitis progression. Prolonged nasal intubation may mechanically obstruct sinus ostia, impair mucociliary clearance, and facilitate bacterial migration from the nasal cavity to the paranasal sinuses.^[12,13]^ This mechanism is consistent with prior reports linking NTT to hospital-acquired sinusitis and subsequent ventilator-associated pneumonia.^[12,14]^ From a clinical perspective, minimizing the duration of NTT, considering orotracheal alternatives, alternating nostrils for prolonged intubation, and implementing routine nasal hygiene measures (e.g., saline irrigation) may help reduce sinus-related complications.^[5]^

Male sex was also independently associated with sinusitis progression. Possible explanations include anatomical differences such as larger sinus volumes with slower drainage, reduced mucociliary transport efficiency, and behavioral factors, including a higher smoking prevalence in male populations.^[14,15]^ Sex-based immunologic differences, particularly in innate and mucosal responses, may also contribute to susceptibility, as reported in studies of upper airway disease.^[10,16]^ Further mechanistic research is needed to clarify the biological basis for this sex disparity.

In Univariable analysis, longer hospital stay correlated with sinusitis progression, but this association was not significant in multivariate modeling. This suggests that hospitalization duration may reflect overall disease severity and the cumulative exposure to invasive interventions, rather than being an independent etiologic factor.^[6,17]^ Identifying direct, modifiable risk factors, such as NTT use, is therefore more clinically meaningful for prevention strategies.

Our findings have several clinical implications. First, routine CT monitoring in high-risk NICU patients, particularly those with unexplained fever, may enable earlier detection of sinus involvement. Second, targeted preventive measures focusing on modifiable risk factors could reduce the incidence of sinusitis and potentially lower the burden of ventilator-associated pneumonia and other secondary infections. Previous interventional studies have shown that early sinus drainage or standardized sinusitis management protocols can shorten ICU stays and improve outcomes.^[12,13]^

This study has several limitations. Its single-center, retrospective design may limit generalizability, as patient demographics, ICU protocols, and intubation practices can vary across institutions. Additionally, the absence of microbiological confirmation prevents definitive pathogen identification and limits the ability to correlate imaging findings with infectious etiology. Although NGT placement was associated with sinusitis progression in univariable group comparisons, it did not remain significant in the multivariable model, suggesting that its effect may be confounded by other clinical factors. Future multicenter, prospective studies incorporating endoscopic evaluation, microbiological sampling, and interventional trials targeting nasal care or intubation strategies are warranted to validate and extend these results.

In conclusion, sinusitis is a frequent and progressive complication in NICU patients, with NTT and male sex identified as independent risk factors. Early recognition may inform strategies to reduce sinus-related complications, which require validation in future prospective studies.

## 5. Conclusion

Nasotracheal intubation and male sex were independently associated with radiologic progression of ICU-acquired sinusitis in neurological patients. Prospective multicenter studies are needed to validate preventive approaches.

## Acknowledgments

The authors would like to thank Fan Su, MS, Department of Biostatistics, Beijing Tiantan Hospital, Capital Medical University, for providing expert consultation and reviewing the statistical analyses for this study.

## Author contributions

**Conceptualization:** Yubin Xue.

**Data curation:** Lin Guan.

**Formal analysis:** Yubin Xue.

**Investigation:** Lin Guan.

**Methodology:** Yubin Xue.

**Project administration:** Jianan Ma, Jianwei Wu.

**Resources:** Yaqiang Zhang.

**Software:** Chao Dong.

**Supervision:** Dong Ma, Jianwei Wu.

**Validation:** Yaqiang Zhang.

**Visualization:** Dong Ma, Chao Dong.

**Writing – original draft:** Yubin Xue.

**Writing – review & editing:** Lin Guan.

## Supplementary Material


