# Supplementary material for: Nasotracheal intubation and male sex as independent risk factors for ICU-acquired sinusitis in neurological patients: A retrospective cohort study
Source: Medicine (Baltimore). 2025 Dec 26;104(52):e46481. doi: 10.1097/MD.0000000000046481 (PMC12747037; doi:10.1097/MD.0000000000046481)
Supplement: Supplementary file 1 [file medi-104-e46481-s001.docx]

# Supplementary Table S1. Surgical treatments of the study cohort (N=250).

| Surgical treatment category | Procedures included | n (%) |
| --- | --- | --- |
| Open cranial surgery | Intracranial hematoma evacuation, decompressive craniectomy, aneurysm clipping/resection | 48 (19.2) |
| Other procedures | Miscellaneous surgical interventions not classified elsewhere | 76 (30.4) |
| Endovascular / interventional procedures | Angiography, endovascular thrombectomy, balloon angioplasty, stenting | 126 (50.4) |

Values are presented as number (percentage) of patients.
Abbreviations: DC, decompressive craniectomy; EVT, endovascular thrombectomy.
